# 基于非靶向代谢组学的尿路上皮癌尿液生物标志物的分析

**DOI:** 10.3724/SP.J.1123.2025.05018

**Published:** 2026-03-08

**Authors:** Sai LIU, Mo WANG, Wei WANG

**Affiliations:** 1.首都医科大学附属北京朝阳医院泌尿外科，北京 100020; 1. Department of Urology，Beijing Chao-Yang Hospital，Capital Medical University，Beijing 100020，China; 2.首都医科大学附属北京朝阳医院，北京 100020; 2. Beijing Chao-Yang Hospital，Capital Medical University，Beijing 100020，China; 3.北京市临床检验中心，北京 100020; 3. Beijing Center for Clinical Laboratory，Beijing 100020，China

**Keywords:** 尿路上皮癌, 尿液, 非靶向代谢组学, 液相色谱-串联质谱, 代谢重编程, urothelial carcinoma （UC）, urine, untargeted metabolomics, liquid chromatography-tandem mass spectrometry （LC-MS/MS）, metabolic reprogramming

## Abstract

尿路上皮癌是全球关注的常见恶性肿瘤之一，缺乏优质的生物标志物。代谢重编程被认为是恶性肿瘤的关键特征。非靶向代谢组学分析可以实现体液样本的高通量检测和无偏向性分析。本研究拟通过非靶向代谢组学方法分析尿路上皮癌患者中特异性代谢产物，筛查潜在的生物标志物。本研究在2020年1~12月于首都医科大学附属北京朝阳医院收集60份尿液样本，其中尿路上皮癌患者30份，健康对照30份。通过结构性表格收集尿路上皮癌患者和健康对照的基本临床资料和尿路上皮癌病理等信息。所有尿液样本均在侵入性操作前予以收集备用。使用四极杆轨道离子阱高分辨质谱仪进行质谱分析，并使用Progenesis QI软件计算质谱信息。尿路上皮癌组和健康对照组的基本临床资料无统计学差异（*P*>0.05）。研究没有在两组的主成分分析图中观察到明显的差异。进一步采用监督性的正交偏最小二乘法判别分析。结果显示，尿路上皮癌患者和健康对照的尿液代谢物差异较大，如L-组氨酸、*N*-乙酰-L-色氨酸和5′-甲硫腺苷等（*P<*0.05）。差异代谢产物涉及多种物质代谢通路，其中组氨酸代谢通路、精氨酸生物合成通路、精氨酸和脯氨酸代谢通路、色氨酸代谢通路等是最主要的，为筛选尿路上皮癌的生物标志物和潜在治疗方法提供了参考。

## Abstract (en)

Urothelial carcinoma （UC） is a globally prevalent malignancy lacking robust non-invasive biomarkers. Metabolic reprogramming is a recognized cancer hallmark. Untargeted metabolomics enables high-throughput and unbiased analysis of bodily fluids， offering a promising approach for discovering novel biomarkers in UC. This investigation employs untargeted metabolomic profiling to detect novel urinary biomarkers in UC cohorts. The analytical strategy prioritizes tumor-associated metabolic perturbations through pathway-centric characterization of dysregulated biochemical networks. This study systematically characterizes differential metabolites and associated pathway dysregulations in UC cohorts. The approach seeks to establish a reliable metabolic signature with diagnostic and prognostic value. The findings are expected to advance the development of novel clinical tools. UC biomarkers should optimally integrate preclinical identification， treatment response tracking， and precision-tailored interventions. This investigation provides a methodological framework for exploring cancer metabolism in UC. And it offers evidence-based insights to support translational research and precision medicine initiatives in oncology. This study was conducted at Beijing Chao-Yang Hospital， Capital Medical University， between January and December 2020. A total of 60 urine specimens were consecutively collected. They comprised 30 histologically confirmed UC patients and 30 healthy controls with normal urinalysis findings. Clinical data was prospectively collected via structured case report forms. It encompassed baseline demographics， comorbidities， anthropometric and behavioral factors， and UC pathological parameters. All urine samples were collected prior to invasive procedures. And they were labeled， snap-frozen in liquid nitrogen， and stored at -80 ℃ until analysis. Metabolic profiling was performed using a quadrupole-orbitrap high resolution mass spectrometer equipped with a heated electrospray ionization source. Mass spectrometric data processing was performed using Progenesis QI software. Data processing followed the following workflow： raw data import， spectral peak alignment， feature extraction， and deconvolution. The Progenesis QI software generated datasets containing retention time， peak intensity， and mass-to-charge ratios. Multivariate signal decomposition enabled independent resolution of adduct species， including protonated and sodium-adducted ions. Quality control measures included elimination of ion features demonstrating intra-batch coefficient of variation >15% across technical replicates. This rigorous preprocessing protocol ensured removal of unstable signals. And it preserved biologically relevant metabolic features for subsequent multivariate analysis. No statistically significant differences were observed in baseline clinical characteristics between UC and healthy control cohorts （*P*>0.05）. Untargeted metabolomic profiling was performed using liquid chromatography-tandem mass spectrometry （LC-MS/MS）. Principal component analysis （PCA） revealed no distinct cluster separation between groups. This result was potentially attributed to limited intergroup metabolic variations or restricted sample size. To enhance discriminatory capacity， supervised orthogonal partial least squares-discriminant analysis （OPLS-DA） was implemented for feature selection. This approach effectively addressed variable collinearity while minimizing non-biological noise interference. Differentially expressed metabolites were identified through variable importance in projection scores， fold change and adjusted P-values. Metabolic pathway analysis was conducted， incorporating pathway enrichment analysis. This multi-tiered analytical approach systematically prioritized UC-associated metabolic perturbations while controlling for confounding factors in specimen analysis. Supervised OPLS-DA was employed to identify differential metabolites and associated metabolic pathways. Significant urinary metabolic disparities were detected between UC patients and healthy controls. Alterations were observed in L-Histidine， *N*-Acetyltryptophan， 5′-methylthioadenosine， *N*-methylnicotinamide， L-octanoylcarnitine， 3-indolehydracrylic acid， *N*¹，*N*¹²-diacetylspermine， pantothenic acid and so on （*P*<0.05）. Pathway enrichment analysis revealed perturbations spanning amino acid metabolism， nucleotide biosynthesis， vitamin cofactor utilization， and carbohydrate processing. The histidine metabolism pathway demonstrated the highest topological impact. It was followed by the arginine biosynthesis pathway， arginine and proline metabolism pathway， and the tryptophan catabolism pathway. Future validation in larger cohorts and mechanistic studies is warranted to confirm their clinical utility. The aberrant pathways may offer novel biomarkers and therapeutic targets， particularly for patients resistant to conventional therapies.

尿路上皮癌（urothelial carcinoma， UC）是全球关注的常见恶性肿瘤之一，2020年全球新诊断的膀胱癌病例超过57万人。预计到2040年，全球膀胱癌新发病例将是2020年的2倍^［1］^。UC起源于泌尿系统黏膜上皮。早期非肌层浸润性膀胱癌若能及时诊断和治疗，治愈率高且患者预后良好。膀胱发生肌层浸润后可能转移到淋巴结、骨骼、肺和肝脏中，中位生存期约15个月^［2，3］^。UC易复发，且晚期预后不良易造成沉重的疾病负担，因而UC的早期筛查和诊断至关重要。UC诊断方法包括尿液细胞学检查、计算机断层扫描和内镜检查。尿液细胞学检查敏感性低，计算机断层扫描存在辐射风险，膀胱镜等内镜检查可能产生疼痛、感染等并发症。UC缺乏无创、快速和精准检测的优质生物标志物。

代谢重编程自系统阐明以来，该现象已被视为恶性肿瘤的关键特征。恶性增殖的癌细胞通过代谢重编程重塑糖、氨基酸和脂质的代谢，满足高速增殖的能量和结构需求。此外，代谢重编程还可以通过凋亡抵抗和免疫逃逸等非代谢效应，营造有利于恶性肿瘤进展的代谢微环境^［4］^。生长因子通过PI3K/AKT通路上调膜转运蛋白介导的糖和氨基酸等营养物质的摄取，为肿瘤增殖提供原料^［5，6］^。生长因子信号进一步激活葡萄糖代谢，并诱导有氧糖酵解，包括直接磷酸化或糖酵解酶的变构调节^［7］^。骨髓细胞瘤病毒癌基因同源物（myelocytomatosis viral oncogene homolog， Myc）直接激活参与嘌呤合成的基因^［8］^。核因子E2相关因子2（nuclear factor erythroid 2-related factor 2， NRF2）是一种关键转录因子，在多种肿瘤中因负反馈丧失而持续激活，激活的NRF2上调丝氨酸生物合成和戊糖磷酸途径的相关基因，从而为核苷酸合成提供了所需的代谢原料^［9］^。甲基化反应依赖甲基供体*S*-腺苷甲硫氨酸，其来源于甲硫氨酸，而甲硫氨酸缺乏可降低体内外细胞组蛋白甲基化的水平^［10］^。代谢重编程导致的代谢物种类和含量差异可以用来区分是否为恶性肿瘤^［11］^。UC的发生和进展同样伴随“代谢重编程”现象^［12，13］^。尿液直接来源于泌尿系统，可以直接反映局部代谢变化。与血液相比，尿液可以无创收集，更易获取，所以更易被大众所接受。因此，通过尿液进行代谢组学分析寻找UC生物标志物具有显著优势^［14］^。

代谢组学通过系统检测生物样本中的代谢物，能够揭示肿瘤细胞代谢重编程特性，这是筛选UC生物标志物的可行方法之一^［15］^。非靶向代谢组学能够对体液样本进行无偏向性的高通量检测，已在生物医学尤其是生物标志物的探索中得到了广泛应用^［16-18］^。尽管已有UC的非靶代谢组学研究，但方法学和结果存在差异^［19，20］^，因此，本研究拟采用非靶向代谢组学方法分析UC患者中特定代谢产物，筛选潜在的生物标志物，以期为科研和临床应用提供依据。

## 1 实验部分

### 1.1 仪器与试剂

Dionex UltiMate 3000超快速液相色谱仪、Q Exactive四极杆轨道离子阱质谱仪和Savant SPD131DDA SpeedVac真空离心浓缩仪（美国Thermo Scientific公司）；Mix-3000振荡混匀器（杭州米欧仪器有限公司）；MIKRO 220R台式高速冷冻离心机（德国Hettich Lab Technology公司）。

甲醇、水和甲酸（色谱纯，美国Thermo Scientific公司）。

### 1.2 人群资料

本研究于2020年1~12月于首都医科大学附属北京朝阳医院开展，研究经过首都医科大学附属北京朝阳医院伦理委员会批准，伦理审批号为（2019-科-320）。通过结构性表格收集UC患者和健康对照的基本临床资料（年龄、性别、高血压、糖尿病、冠心病、体重身高指数（body mass index， BMI）、吸烟和饮酒情况）和UC病理信息（分期和分级）等。本研究共收集60份尿液样本，其中UC患者30份，健康对照30份。

### 1.3 样本处理

所有尿液样本均在侵入性操作前予以收集，标记后经液氮冷冻，储存于-80 ℃冰箱中备用。实验开始前取出所有样本，并置于4 ℃下解冻，涡旋10 s混匀。取150 μL尿液样本，置于1.5 mL离心管中，其内加入150 μL甲醇，振摇1 min。然后，将样本离心管置于低温离心机内，于4 ℃和12 000 r/min下离心10 min。取上清液120 μL，置于液相色谱小瓶（内置250 μL内插管）中，备用。

质控样本由等体积的各尿液样本均匀混合配制得到，质控样本的检测和数据采集等与其他样本完全一致。

### 1.4 分析条件

#### 1.4.1 色谱条件

色谱柱：Waters UPLC HSS T3（100 mm×2.1 mm，1.8 μm）；柱温：50 ℃；流速0.3 mL/min；进样量：4 μL。流动相A：0.1%甲酸水溶液，流动相B：甲醇。梯度洗脱程序：0～1.0 min，2%B；1.0～5.5 min，2%B～100%B；5.5～14.0 min，100%B；14.0～14.1 min，100%B～2.0%B；14.1～16.0 min，2%B。

#### 1.4.2 质谱条件

使用四极杆轨道离子阱高分辨质谱仪（具有热电喷雾离子源）进行质谱分析。毛细管设置温度：320 ℃；正离子的离子源电压：3.7 kV；负离子的离子源电压为-3.5 kV；鞘气压力：206 843 Pa，辅助气压力：68 948 Pa；鞘气：氮气；辅助气：氮气；碰撞气：氮气，压力为0.2 Pa；溶剂加热蒸发温度：300 ℃。一级全扫描参数：分辨率70 000，最大隔离时间50 ms，自动增益控制目标1×10^6^。使用外标法校准质谱质量轴，质量误差：5×10^-6^。代谢物鉴定参数如下：采用数据依赖扫描模式（dd-MS^2^扫描模式），分辨率17 500，最大隔离时间50 ms，自动增益控制目标1×10^5^，最多扫描10个离子的二级碎片（动态排除），质量分离窗口2，碰撞能：10、30、60 V。

### 1.5 数据分析

本研究使用Progenesis QI软件计算质谱信息，处理顺序为录入原始数据、质谱峰对齐、质谱峰提取、去卷积，然后产生包括保留时间、峰强度和质荷比的数据。通过多组分信号解析实现加合离子（例如加氢、加钠等）信息各自独立。样本中变异系数>15%的离子信息被视为不稳定数据，均被去除。本研究采用描述性统计学方法对研究人群的基本临床资料和UC病理信息进行分析，连续变量采用平均值±标准差来表示，分类资料采用数值（%）表示。

使用主成分分析（principal component analysis， PCA）对包括质控样品在内的所有样品进行分析。质控信号为多次进样的同一样品经检测所获取的信号，这些信号经过处理后应当在PCA图中重叠。如重叠良好，则表明检测和处理系统均稳定，研究的数据可以进一步分析。

对所有样本进行PCA分析能从全局上展示样本之间的代谢变异度。在使用Simca 14.1软件正式分析前，对质谱数据进行处理，确保数据的一致性，从而得到清晰和稳定的结果。质谱数据的目的是使全部变量处于同一等级上，通过动态范围调节技术消除代谢物浓度梯度引起的信号遮蔽，确保均一性。PCA是非监督性模型分析，若区分样本代谢变异能力较差，为了进一步取得显著差异的代谢物信息，我们采用监督性的正交偏最小二乘方判别分析（orthogonal partial least squares discriminant analysis， OPLS-DA）对两组尿液样本进行统计分析。

本研究使用OPLS-DA模型的重要变量投影（VIP）值（阈值>1），变化倍数（fold change， FC）>1和*t*-test的*P*值（*P<*0.05）来筛选差异代谢物。差异代谢物的定性方法如下：通过搜索网络数据库（如HMDB和LIPID MAPS）和自建化合物库，核对本研究结果和数据库筛选物质的*m/z*或者精确分子质量，误差限制为5×10^-6^。

## 2 结果与讨论

### 2.1 基本临床资料和UC病理信息

UC患者和健康对照的基本临床资料和UC病理信息如表1所示。UC患者和健康对照的平均年龄是（55.9±10.2） 岁和（53.1±9.8） 岁，两组在年龄、性别、BMI、高血压、糖尿病、冠心病、吸烟和饮酒等各项指标中均无统计学差异。UC患者中16例低级别乳头状尿路上皮癌（low-grade papillary urothelial carcinoma， LGPUC）和14例高级别乳头状尿路上皮癌（high-grade papillary urothelial carcinoma， HGPUC）；UC患者中分期原位癌（carcinoma in situ， Tis）1例，非浸润性乳头状癌（papillary non-invasive carcinoma， Ta）9例，肿瘤侵犯上皮下结缔组织（tumor invades subepithelial connective tissue， T1）8例，肿瘤侵犯肌层（tumor invades muscularis propria， T2）12例。

**表1:** 基本临床资料和尿路上皮癌病理信息

Parameter	Total （*n*=60）	UC （*n*=30）	HC （*n*=30）	*P*
Age/years	54.5±10.0	55.9±10.2	53.1±9.8	0.282
Gender （male/female）	34/26	18/12	16/14	0.795
BMI/（kg/m^2^）	23.2±2.7	23.6±2.6	22.8±2.7	0.247
Hypertension^*^	28 （46.7%）	15 （50.0%）	13 （43.3%）	0.796
Diabetes mellitus^*^	29 （48.3%）	14 （46.7%）	15 （56.7%）	0.606
Coronary artery disease^*^	18 （30.0%）	8 （26.7%）	10 （33.3%）	0.779
Smoking consumption status^*^	39 （65.0%）	21 （70.0%）	18 （60.0%）	0.589
Alcohol consumption status^*^	21 （35.0%）	6 （20.0%）	15 （26.7%）	0.761
LGPUC^*^		16 （53.3%）		
HGPUC^*^		14 （46.7%）		
Tis^*^		1 （0.03%）		
Ta^*^		9 （30.0%）		
T1^*^		8 （26.7%）		
T2^*^		12 （40.0%）		

UC： urothelial carcinoma； HC： healthy controls； BMI： body mass index； LGPUC： low-Grade papillary urothelial carcinoma； HGPUC： high-grade papillary urothelial carcinoma. Quantitative outcomes were expressed as mean±SD. *P* values were evaluated by *t*-tests. Tis： carcinoma in situ； Ta： papillary non-invasive carcinoma； T1： tumor invades subepithelial connective tissue； T2： tumor invades muscularis propria； * number of patients or healthy controls and the corresponding percentage.

### 2.2 尿液代谢物检测和质量控制

图1a和图1b分别展示了本研究在正、负离子模式下获得的代表性色谱图。我们建立了尿液样本的PCA模型，如图1c和图1d所示，UC表示UC患者尿液样本，QC表示质控样本，QC样本重叠紧密，表示样本处理和数据分析不存在仪器漂移或批次效应等情况，实验结果呈现优良的稳定性和可靠性。

**图1:**
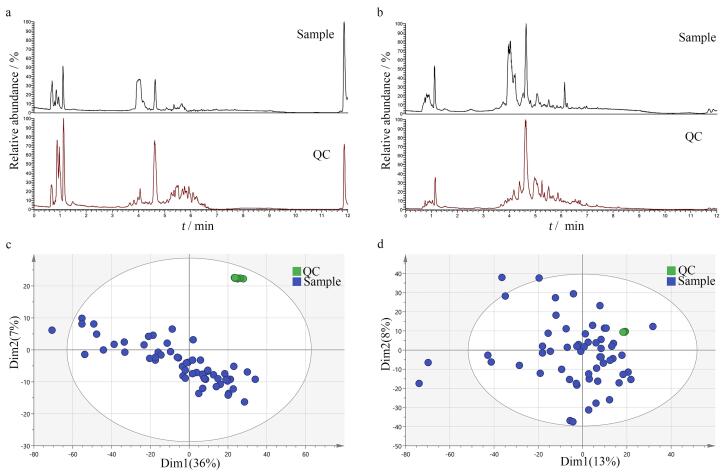
尿液样本在（a、c）正、（b、d）负离子模式下的代表性色谱图和PCA得分图

#### 2.2.1 尿液代谢物的多变量分析

为筛选UC患者和健康对照尿液中差异代谢物，初步采用了PCA模型进行尿液样本分析。但是未观察到两组样本明显的区分，可能与差异代谢产物较少或样本量较小等因素相关。

为进一步识别差异代谢物，我们采用了OPLS-DA分析两组尿液代谢物，结果如图2所示。UC患者和健康对照尿液样本的代谢谱通过OPLS-DA分析得到了显著区分。我们对该分析结果进行了多次重复，OPLS-DA分析结果具有良好的稳定性和可靠性。

**图2:**
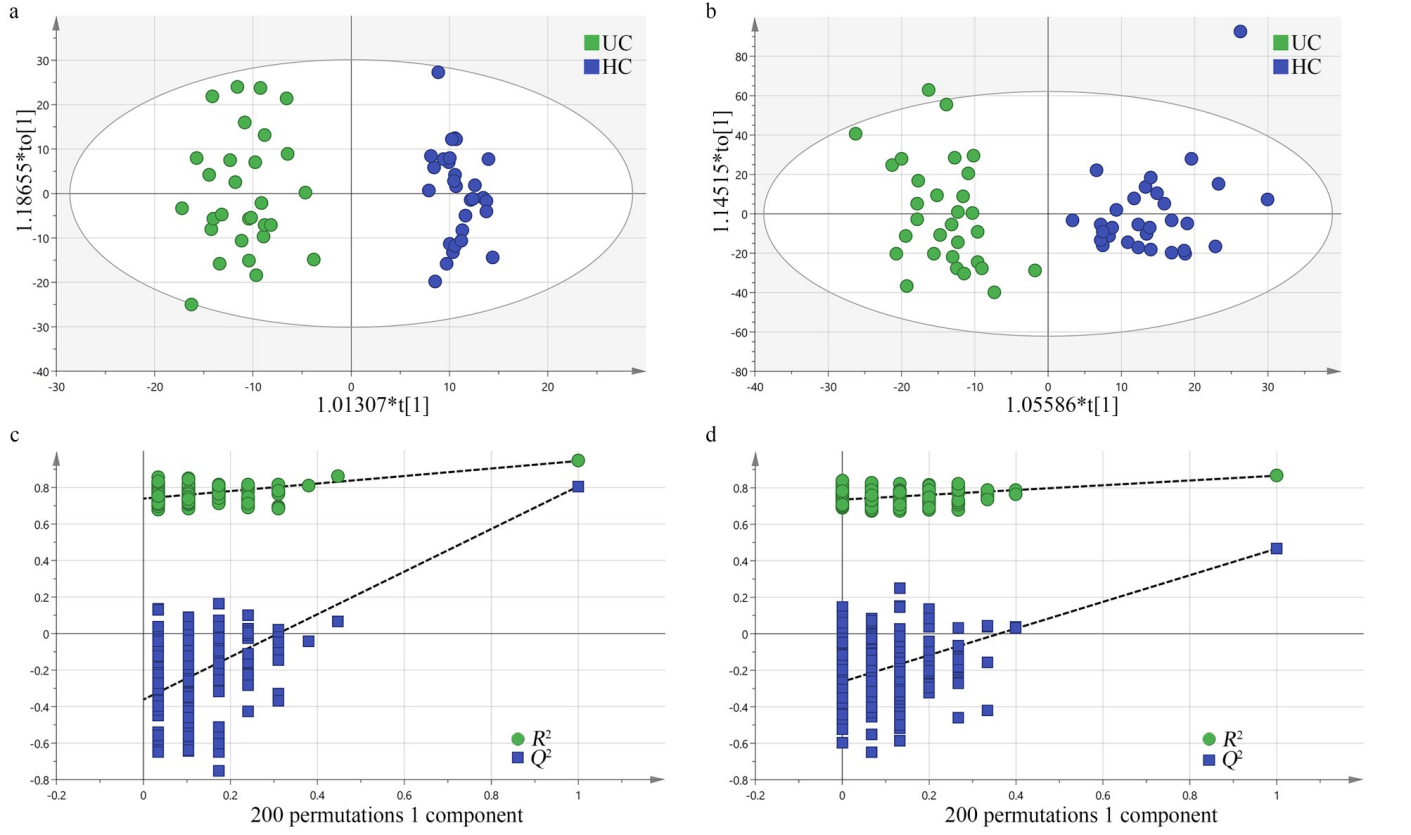
尿路上皮癌组和健康对照组在（a、c）正、（b、d）负离子模式下的OPLS-DA分析结果和置换检验结果

#### 2.2.2 差异代谢物和相关代谢通路

如图3所示，UC患者和健康对照按FC、VIP和*P*值筛选后，得到差异代谢物，如L-组氨酸、*N*-乙酰-L-色氨酸、5′-甲硫腺苷、*N*-甲基烟酰胺、辛酰-L-肉碱、吲哚-3-丙烯酸、*N*¹，*N*¹²-二乙酰精胺和泛酸等。我们对差异代谢物进行分析，寻找涉及的代谢通路，如图4和表2所示。根据分析结果，涉及氨基酸、核苷酸、维生素和糖代谢等代谢通路，其中氨基酸代谢通路的种类和差异值等在所涉及的通路中均是最主要或最显著的。每个代谢通路涉及众多物质，而代谢通路间又存在交叉与共用中间产物，构成了包括UC在内的恶性肿瘤的复杂代谢通路。

**图3:**
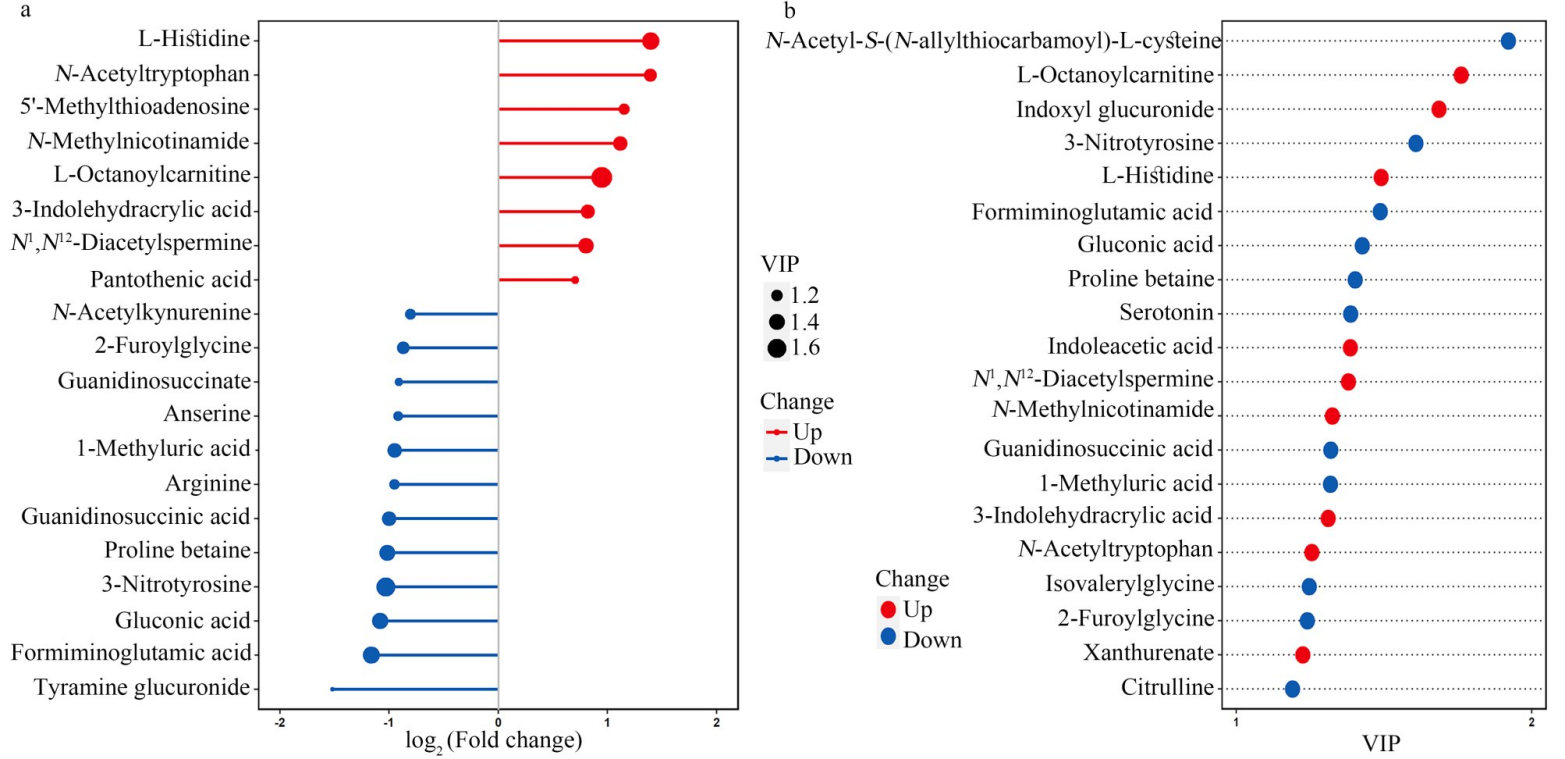
尿路上皮癌组和健康对照的（a）变化倍数和（b）重要变量投影值排名前20的尿液差异代谢物

**图4:**
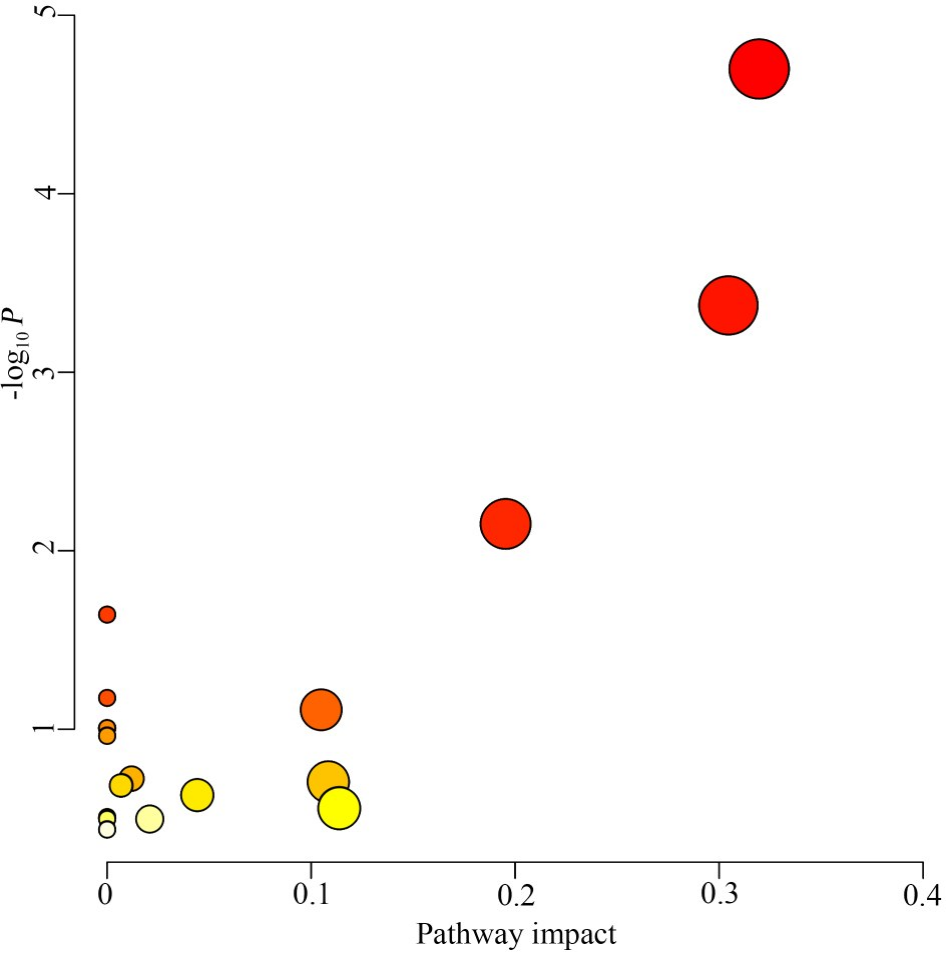
尿路上皮癌组和健康对照组的通路分析

**表2:** 尿路上皮癌组和健康对照组尿液差异代谢产物的通路富集结果

Pathway name	-log_10_ *P*	Impact
Histidine metabolism	4.6988	0.31967
Arginine biosynthesis	3.3744	0.30457
Arginine and proline metabolism	2.1504	0.19535
*β*-Alanine metabolism	1.6423	0
Nitrogen metabolism	1.1756	0
Tryptophan metabolism	1.1091	0.10493
Ascorbate and aldarate metabolism	1.0065	0
Vitamin B6 metabolism	1.0065	0
Caffeine metabolism	0.96305	0
Purine metabolism	0.72364	0.01196
Pentose and glucuronate interconversions	0.70508	0.10843
Pantothenate and CoA biosynthesis	0.68509	0.0068
Pentose phosphate pathway	0.63125	0.04419
Alanine， aspartate and glutamate metabolism	0.55718	0.11378
Glyoxylate and dicarboxylate metabolism	0.50821	0
Glycine， serine and threonine metabolism	0.49709	0
Cysteine and methionine metabolism	0.49709	0.02089
Pyrimidine metabolism	0.43795	0

在UC的发生和进展过程中，氨基酸不仅为UC增殖提供所需的原料，同时还参与免疫调节、能量代谢和遗传表达调控等。如图4和表2所示，氨基酸代谢主要富集于组氨酸代谢通路、精氨酸生物合成通路、精氨酸和脯氨酸代谢通路、色氨酸代谢通路等。

组氨酸和精氨酸虽然能够体内合成，一般被认为是非必需氨基酸，但是在婴幼儿、慢病消耗或疾病恢复等身体状态下，内源合成不足，需要外源性补充，所以又被称为条件必需氨基酸。组氨酸的咪唑基团可发生甲基化或质子化修饰，进而改变蛋白质结构和功能^［21］^。在人体中，组氨酸深度参与人体内很多的生命活动。组氨酸在脱羧酶催化后转化为组胺，组胺能够舒张血管，调节血管壁的通透性，参与变态反应。组胺逐级酶解后转化为咪唑乙酸等产物。肝癌的发生常伴随周围免疫微环境的变化，组氨酸代谢物如组胺在恶性肿瘤的炎症反应中起着关键作用。组胺通过其受体（例如H1、H2、H3和H4受体）调节免疫细胞功能，包括T细胞、B细胞、巨噬细胞和树突状细胞，从而影响肿瘤的免疫微环境^［22］^。组氨酸代谢与氧化应激的调节密切相关，可能通过维持抗氧化状态促进肿瘤细胞存活。肝癌细胞经常处于高氧化应激状态，这同样促进了肿瘤细胞的存活和增殖^［23］^。组氨酸代谢与肝癌细胞的能量需求密切相关。组氨酸通过生成谷氨酸参与三羧酸循环，从而影响肝细胞的能量供应。在肝癌中，代谢重编程驱动了更高的能量需求，组氨酸代谢可能通过调节能量代谢途径影响肿瘤的增殖和生存^［24］^。综上，组氨酸代谢在肿瘤免疫调节和代谢重编程中发挥多重作用，可能在UC中具有类似机制。并且，组氨酸代谢驱动肝恶性肿瘤发生代谢重编程，并且通过细胞间信号通路影响肿瘤免疫微环境，从而促进恶性肿瘤增殖^［25］^。

精氨酸的侧链带正电荷，可以作为蛋白质的亲水端结合带有负电荷的分子。精氨酸是一种多功能的氨基酸，除了作为蛋白质合成的基本成分外，它还是多胺、肌酸和一氧化氮的前体，并深度参与尿素循环。精氨琥珀酸合成酶为精氨酸合成的限速酶，其表达在多种恶性肿瘤中发生变化。它在某些癌症中过表达，包括结肠癌、肺癌、胃癌和卵巢癌，但在其他癌症如肾细胞癌、黑色素瘤、前列腺癌和肝细胞癌中则可能丢失^［26］^。精氨酸还可以与脯氨酸和谷氨酸相互转化，并通过或不通过哺乳动物雷帕霉素靶蛋白复合物1（mammalian target of rapamycin complex 1，mTORC1）两种途径影响恶性肿瘤的代谢^［27］^。在肝癌中，研究人员发现尽管精氨酸合成受到抑制，但由于精氨酸输入增加和精氨酸转化为多胺的减少，肝癌中的精氨酸水平仍然升高。高浓度的精氨酸重新编程代谢以促进肿瘤发生。精氨酸结合RNA结合基序蛋白39促进天冬酰胺的合成。天冬酰胺进一步增强精氨酸的摄取，形成正反馈循环，维持高水平的精氨酸和致癌代谢^［28］^。精氨酸代谢通过各种细胞之间复杂的代谢反应，重塑肿瘤微环境，驱动恶性肿瘤进展。例如，在乳腺癌的肿瘤微环境内，精氨酸会诱导肿瘤相关巨噬细胞的亲瘤极化，从而抑制T细胞的抗肿瘤活性。但是，癌细胞-巨噬细胞介导的肿瘤促进作用超过了精氨酸介导的T细胞抗肿瘤作用。精氨酸代谢产物多胺通过胸腺嘧啶DNA糖基酶介导的DNA去甲基化，增强促肿瘤相关巨噬细胞极化。重要的是，针对癌细胞与巨噬细胞之间的精氨酸-多胺代谢轴显著抑制乳腺癌生长，突显其治疗潜力^［29］^。

脯氨酸是唯一在吡咯烷环中具有*α*-氨基的亚氨基酸，常位于肽链的转角处，影响蛋白质的二级结构。脯氨酸的代谢产物*γ*-氨基丁酸可以降低炎症因子水平，改善炎症状态。脯氨酸能用于合成胶原蛋白，而胶原蛋白是细胞外基质的主要成分。脯氨酸相关代谢酶在癌症中拥有诸多重要功能。脯氨酰4-羟化酶（prolyl 4-hydroxylase， P4H）存在于内质网中，P4HB在膀胱癌、结肠癌和肝细胞癌中过表达，在胶原蛋白的生物合成中起着核心作用。在膀胱癌中，抑制P4HB可减少细胞增殖，促进细胞凋亡，并通过激活内质网应激和凋亡途径使癌细胞对吉西他滨更加敏感。在结肠癌中，敲除P4HB可以促进活性氧积累并失活信号转导子和转录激活子3信号通路诱导细胞凋亡。癌细胞中脯氨酸代谢与癌细胞的增殖、转移和药物耐受有关。缺氧状态可以诱导脯氨酸合成，导致羟基脯氨酸累积，从而促进肝癌的发生和索拉非尼的耐药性。这些研究表明，脯氨酸代谢酶参与了多种肿瘤疾病过程。充分研究脯氨酸代谢途径将为癌症诊断和治疗提供新的策略和思路^［30］^。

色氨酸为必需氨基酸，由膳食摄取，侧链结构大，呈弱极性或无极性，经常位于水溶性蛋白质的内部，能够转化成神经递质5-羟色胺。色氨酸对蛋白质生物合成至关重要，并且色氨酸是多种关键化合物的前体。色氨酸的代谢途径主要包括3条：第一条是通过犬尿氨酸途径氧化色氨酸，大约95%的色氨酸通过此途径被代谢。该途径与抑郁症、功能性胃肠疾病、免疫紊乱、心血管疾病、神经退行性疾病和肿瘤的发展有关。第二条途径是通过血清素途径羟化色氨酸，这条途径在肠脑轴中起着关键作用，并与多种神经精神障碍相关；研究表明，血清素具有抗炎特性，提供神经保护作用。然而，在各种癌症中，它可能通过促进血管生成和调节免疫系统来促进癌症进展。第三条途径是色氨酸通过肠道菌群代谢为吲哚-3-丙酸，吲哚-3-丙酸对恶性肿瘤可能同时存在抗肿瘤和促肿瘤双重作用^［31］^。

犬尿氨酸途径和吲哚途径是色氨酸主要的代谢途径，广泛参与消化、神经和泌尿系统疾病的发生和发展。在犬尿氨酸途径中至关重要的酶，如吲哚胺-2，3-双加氧酶和色氨酸-2，3-双加氧酶，通过耗尽色氨酸，或通过其代谢物激活芳香烃受体，在肿瘤及邻近淋巴结中诱发肿瘤免疫抵抗。吲哚胺-2，3-双加氧酶可以通过非酶途径影响免疫反应。犬尿氨酸途径通过促进血管生成、增强肿瘤转移以及抑制肿瘤铁死亡等机制，参与肿瘤进展。吲哚途径中的吲哚及其相关代谢物参与胃肠道稳态、肿瘤免疫和药物耐受。吲哚代谢产物和相关肠道微生物群在决定肿瘤治疗策略方面起着关键作用，并可能影响免疫化疗的效果。值得注意的是，犬尿氨酸途径和吲哚途径对肿瘤表型的影响可能存在双向性，提示在肿瘤不同类型或阶段中的作用较为复杂。差异代谢通路的关键物质可能为筛选生物标志物或治疗方法提供潜在机会^［31］^。

## 3 结论

本研究基于液相色谱-串联质谱平台，对UC患者和健康对照的尿液样本完成了非靶向代谢组学分析。分析结果显示，UC患者和健康对照的尿液代谢物差异较大，涉及氨基酸、核苷酸、维生素和糖代谢等多种物质代谢通路，其中组氨酸代谢通路、精氨酸生物合成通路、精氨酸和脯氨酸代谢通路、色氨酸代谢通路等在所涉及的通路中均是最主要或最显著的，为筛选UC的生物标志物和潜在治疗方法提供了参考。

需要注意的是，本研究存在一些局限性。本研究基于尿液样本，未结合血液和组织代谢组数据，可能未能全面反映UC和健康对照的代谢差异。本研究局限于非靶向代谢组学对尿液样本进行定性或半定量，未来结合靶向代谢组学精准定量可以为UC的诊疗提供更多的依据。
